# Emergency and Elective Colorectal Cancer—Relationship between Clinical Factors, Tumor Topography and Surgical Strategies: A Cohort Study

**DOI:** 10.3390/medicina60060898

**Published:** 2024-05-29

**Authors:** Ionuţ Simion Coman, Raluca Cristina Vital, Violeta Elena Coman, Cosmin Burleanu, Mircea Liţescu, Costin George Florea, Daniel Alin Cristian, Gabriel-Petre Gorecki, Petru Adrian Radu, Iancu Emil Pleşea, Anwar Erchid, Valentin Titus Grigorean

**Affiliations:** 1 10th Clinical Department—General Surgery, Discipline of General Surgery—“Bagdasar-Arseni” Clinical Emergency Hospital, Faculty of Medicine, “Carol Davila” University of Medicine and Pharmacy, 37 Dionisie Lupu Street, 020021 Bucharest, Romania; ionut.coman@umfcd.ro (I.S.C.); elena.coman@umfcd.ro (V.E.C.); valentin.grigorean@umfcd.ro (V.T.G.); 2 General Surgery Department, “Bagdasar-Arseni” Clinical Emergency Hospital, 12 Berceni Road, 041915 Bucharest, Romania; ralumateescu1310@gmail.com (R.C.V.); burleanucosmin@gmail.com (C.B.); costinflorea1990@gmail.com (C.G.F.); erchid.anwar@yahoo.com (A.E.); 3 2nd Department of Surgery and General Anesthesia, Discipline of Surgery and General Anesthesia—“Sf. Ioan” Clinical Emergency Hospital, Faculty of Dental Medicine, “Carol Davila” University of Medicine and Pharmacy, 37 Dionisie Lupu Street, 020021 Bucharest, Romania; 4 General Surgery Department, “Sf. Ioan” Clinical Emergency Hospital, 13 Vitan-Bârzeşti Road, 042122 Bucharest, Romania; 5 10th Clinical Department—General Surgery, Discipline of General Surgery—“Colţea” Clinical Hospital, Faculty of Medicine, “Carol Davila” University of Medicine and Pharmacy, 37 Dionisie Lupu Street, 020021 Bucharest, Romania; daniel.cristian@umfcd.ro; 6 General Surgery Department, “Colţea” Clinical Hospital, 1 Ion C. Brătianu Boulevard, 030167 Bucharest, Romania; 7 Faculty of Medicine, “Titu Maiorescu” University, 67A Gheorghe Petraşcu Street, 031593 Bucharest, Romania; gabriel.gorecki@prof.utm.ro; 8 Department of Anesthesia and Intensive Care, CF2 Clinical Hospital, 63 Mărăşti Boulevard, 011464 Bucharest, Romania; 9 10th Clinical Department—General Surgery, Discipline of General Surgery—“Dr. Carol Davila” Clinical Nephrology Hospital, Faculty of Medicine, “Carol Davila” University of Medicine and Pharmacy, 37 Dionisie Lupu Street, 020021 Bucharest, Romania; petru.radu@umfcd.ro; 10 General Surgery Department, “Dr. Carol Davila” Clinical Nephrology Hospital, 4 Griviţei Road, 010731 Bucharest, Romania; 11 Pathology Department, “Bagdasar-Arseni” Clinical Emergency Hospital, 12 Berceni Road, 041915 Bucharest, Romania; pie1956@yahoo.com

**Keywords:** colorectal, cancer, emergency, analysis, outcome

## Abstract

*Background and Objectives*: The purpose of the study was to analyze the relationships among several clinical factors and also the tumor topography and surgical strategies used in patients with colorectal cancer. *Materials and Methods:* We designed an analytical, observational, retrospective study that included patients admitted to our emergency surgical department and diagnosed with colorectal cancer. The study group inclusion criteria were: patients admitted during 2020–2022; patients diagnosed with colorectal cancer (including the ileocecal valve); patients who benefited from a surgical procedure, either emergency or elective. *Results*: In our study group, consisting of 153 patients, we accounted for 56.9% male patients and 43.1% female patients. The most common clinical manifestations were pain (73.2% of the study group), followed by abdominal distension (69.3% of the study group) and absence of intestinal transit (38.6% of the study group). A total of 69 patients had emergency surgery (45.1%), while 84 patients (54.9%) benefited from elective surgery. The most frequent topography of the tumor was the sigmoid colon, with 19.60% of the patients, followed by the colorectal junction, with 15.68% of the patients, and superior rectum and inferior rectum, with 11.11% of the patients in each subcategory. The most frequent type of procedure was right hemicolectomy (21.6% of the study group), followed by rectosigmoid resection (20.9% of the study group). The surgical procedure was finished by performing an anastomosis in 49% of the patients, and an ostomy in 43.1% of the patients, while for 7.8% of the patients, a tumoral biopsy was performed. *Conclusions*: Colorectal cancer remains one of the most frequent cancers in the world, with a heavy burden that involves high mortality, alterations in the quality of life of patients and their families, and also the financial costs of the medical systems.

## 1. Introduction

Colorectal cancer (CRC) is a malignancy of the gastrointestinal tract that arises either from the colon or the rectum. Although colon cancer and rectal cancer can be defined as separate diseases, common biological and clinical features determine the study of colorectal cancer as a self-standing entity [1].

The epidemiology of colorectal cancer varies significantly between different areas in the world and also between different gender, racial, and age groups [2]. The highest incidence rates for the year 2020 were in Australia/New Zealand and European regions (40.6 per 100,000 males) and the lowest in Southern Asia and several African regions (4.4 per 100,000 females). The highest mortality was observed in Eastern Europe (20.2 per 100,000 males) and the lowest was observed in South Asia (2.5 per 100,000 females) [3]. 

Multiple factors have been demonstrated to be implicated in the development of colorectal cancer. They include family and personal medical history (family history and genetics, inflammatory bowel disease, cholecystectomy, and diabetes mellitus), lifestyle risk factors (obesity, physical inactivity, cigarette smoking, alcohol consumption, and several dietary patterns such as a diet high in red and processed meat, a diet low in fiber, fruit, and vegetables, or a diet low in calcium, vitamin D, and dairy products) or various other factors (age, gut microbiota, gender, race, or socioeconomic factors) [4]. In addition, it is well-accepted that the majority of colorectal carcinomas evolve from adenomatous polyps. The sequence of events leading to this transformation is known as the adenoma-to-carcinoma sequence [5]. 

Surgical treatment for colorectal cancer can be classified as elective or emergency. While the majority of colorectal cancer presents as elective, an important percentage, around 10–30%, presents as an emergency [6,7]. This rate of emergency remains high, despite the efforts of many countries to implement a colorectal cancer screening program [8]. An association has been proven between emergency presentations of colorectal cancer and significant short-term and long-term outcomes. 

Recent research studies suggest that emergency presentation is an independent poor prognostic indicator following curative colorectal resection [9,10]. These differences in outcomes regarding patients with emergency presentations compared to elective presentations are attributed to differences regarding tumor development and patient-related factors rather than emergency presentation per se [8]. 

Therefore, our objective was to analyze the relationships among several factors, but also the tumor topography and surgical strategies used in patients with colorectal cancer, presenting both as emergency or elective surgeries. 

## 2. Materials and Methods

### 2.1. Study Design

We designed an analytical, observational, retrospective study that included patients admitted to our emergency surgical department and diagnosed with colorectal cancer. Being a retrospective study, statistical power was used instead of the calculation of a sample size. Our study group consisted of patients who met all the inclusion criteria, and comprised 153 patients. 

### 2.2. Inclusion and Exclusion Criteria

The inclusion criteria were:

- patients admitted to our surgical department during 2020–2022 (three years);
- patients diagnosed with colorectal cancer (including the ileocecal valve);
- patients who benefited from a surgical procedure, either emergency or elective.

The exclusion criteria consisted of:

- patients with benign lesions in the colorectal segment;
- patients admitted with the diagnosis of colorectal cancer but who did not benefit from a surgical procedure or refused it;
- incomplete data were recovered from the patients’ files—when we could not obtain all the specific variables from our study.

### 2.3. Ethical Considerations 

All the patients in the study signed a consent form agreeing to be included in a scientific study. This consent form had been approved by the Ethics Committee of the “Bagdasar-Arseni” Clinical Emergency Hospital and the Manager of the “Bagdasar-Arseni” Clinical Emergency Hospital by decision number 537/06.07.2020. 

### 2.4. Statistical Analysis

Data were obtained from the operative protocols and the patients’ files as various nominal and scale variables that compiled our database in Microsoft Office Excel version 2019^®^ software.

We used the following variables:

- scale variables: age, days of hospitalization, white blood cell value, hemoglobin value;
- nominal variables: gender, type of presentation (emergency/elective), area of origin of the patient, clinical manifestations on admittance, presence of a Computer Tomography scan, topography of the tumor, type of surgical procedure that was performed, postoperative complications, mortality.

Depending on the type of explanatory or outcome variables, or the distribution (Gaussian/non-normal) of scale variables, we used parametric or non-parametric tests to evaluate the null hypothesis that we formulated for each association. 

The results were analyzed using IBM SPSS Statistics version 20^®^ software and were integrated into the clinical context.

## 3. Results

Our study group consisted of 153 patients, divided into 87 male patients (56.9%) and 66 female patients (43.1%). 

**Figure 1:**
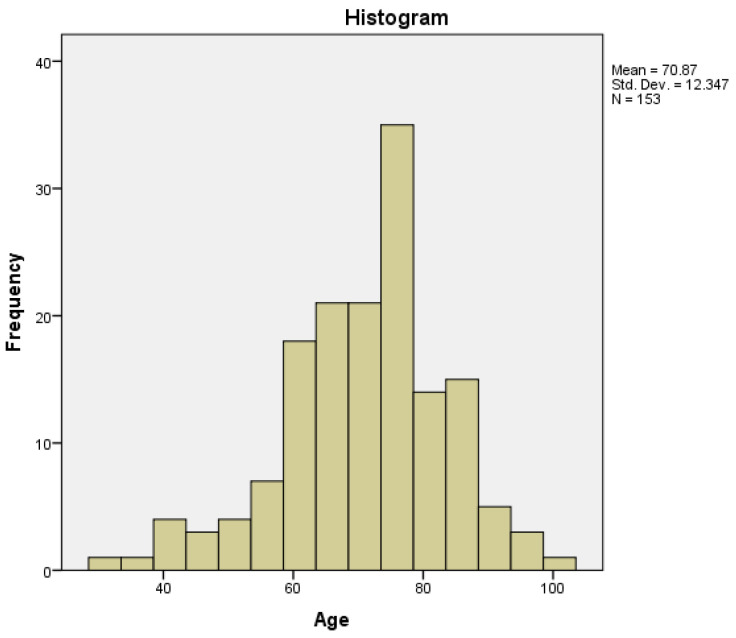
Age distribution in the study group.

The minimum age was 31 years and the maximum was 100 years, with a mean of 70.87 years and a median of 72 years (95% CI (confidence interval)). The age distribution had a normal (Gaussian) distribution (histogram—Figure 1), with a skewness of −0.548, a kurtosis of 0.582, and a *p*-value of 0.026 obtained by a Kolmogorov–Smirnov normality test. 

**Figure 2:**
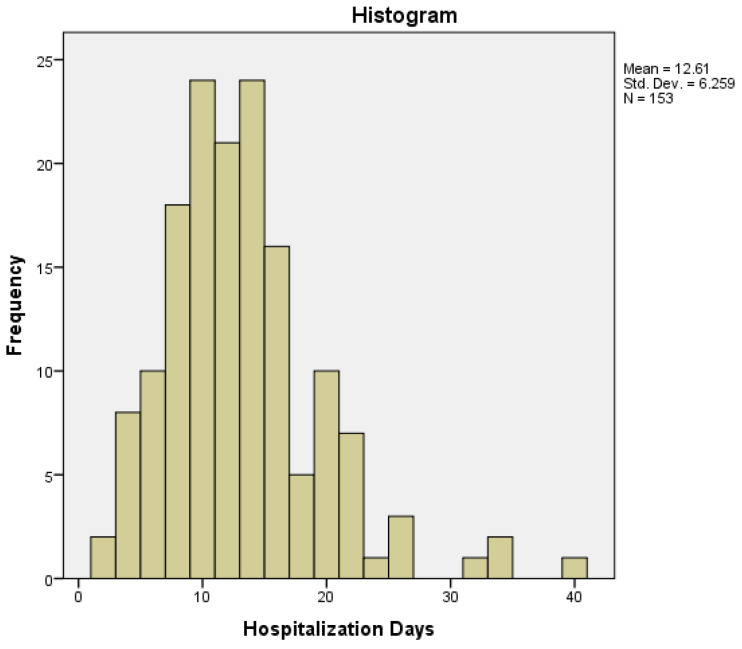
Hospitalization days distribution in the study group.

The minimum number of hospitalization days was two days, while the maximum number was 39 days, with a mean of 12.61 days, a median of 11 days (95% CI), and an interquartile range of 7. The distribution of hospitalization days was non-normal (histogram—Figure 2), with a moderate skewness of 1.236 (tail to the right), a kurtosis of 2.726, and a *p*-value < 0.001 obtained by a Kolmogorov–Smirnov normality test. 

Regarding the distribution of age among the two genders, the minimum age for women was 36 years, while the maximum was 100 years. The mean value was 70.85 years, the median value was 72.50 years, and the distribution was normal, with a skewness of −0.420, a kurtosis of 0.143, and a *p*-value for the Kolmogorov–Smirnov normality test of 0.2. For the male population, the minimum value was 31 years, while the maximum was 94 years. The mean value was 70.89 years, the median value was 72 years, and the distribution was also normal, with a skewness of −0.719, a kurtosis of 1.094, and a *p*-value for the Kolmogorov–Smirnov normality test of 0.66. Therefore, we performed a parametric two-sample *t*-test that obtained a *p*-value of 0.986, with no statistical significance between the two genders regarding age.

The duration of hospitalization between the genders had a minimum value of two days for men and three days for women, while the maximum value was 39 days for men and 26 days for women. The mean values were 13.03 days for men and 12.06 for women, while the distribution was non-normal for men. Therefore, we determined the median values of both categories, with a median value of 12 days for men and respectively of 11 days of women, both of them with an interquartile range of 7. We performed a non-parametric Mann–Whitney U test, obtaining a *p*-value of 0.717; therefore, the distribution of hospitalization days was the same across both genders, with no statistical significance.

A total of 99 patients (64.7% of the study group) came from urban areas, while 54 patients (35.3% of the study group) came from rural areas. There was no significant difference regarding gender and area of provenance (chi-square test, *p* = 0.865). The mean age of patients from urban areas was 71.74, while the mean age for patients from rural areas was 69.28. The mean number of hospitalization days for patients from urban areas was 11.94 days, while the mean number of hospitalization days for patients from rural areas was 13.85 days. There was no correlation between the area of provenance and the age of the patients (two-sample *t*-test, *p* = 0.240) or the length of hospitalization (Mann–Whitney U test, *p* = 0.187).

In our study group, 69 patients received emergency surgery (45.1%), while 84 patients (54.9%) benefited from elective surgery. The mean value for age regarding the patients receiving emergency surgery (71.25 years) was similar to the mean value of the age of the patients operated electively (70.56 years). Regarding hospitalization days, the median value for emergency patients (12 days) was similar to the median value for elective patients (11 days).

The relationships among the emergency/elective presentation of the patient and several other parameters (age, hospitalization days, gender and the area of origin—urban/rural) were analyzed, but no statistical associations were found (Table 1).

**Figure 3:**
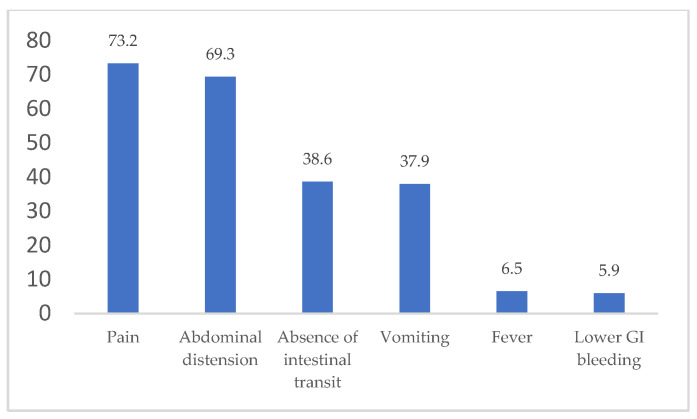
Distribution of clinical manifestations of the study group on admittance (%).

The clinical manifestations of admittance to our general surgery department included pain, nausea/vomiting, abdominal distension, absence of intestinal transit, lower GI (gastrointestinal) bleeding, and fever/shivers (Figure 3). 

The most common clinical manifestations were represented by pain—112 patients (73.2% of the study group), followed by abdominal distension—106 patients (69.3% of the study group) and absence of intestinal transit—59 patients (38.6% of the study group).

An abdominal CT (Computer Tomography) scan was performed preoperatively in 85 patients (55.6% of the study group) (Table 2). 

**Figure 4:**
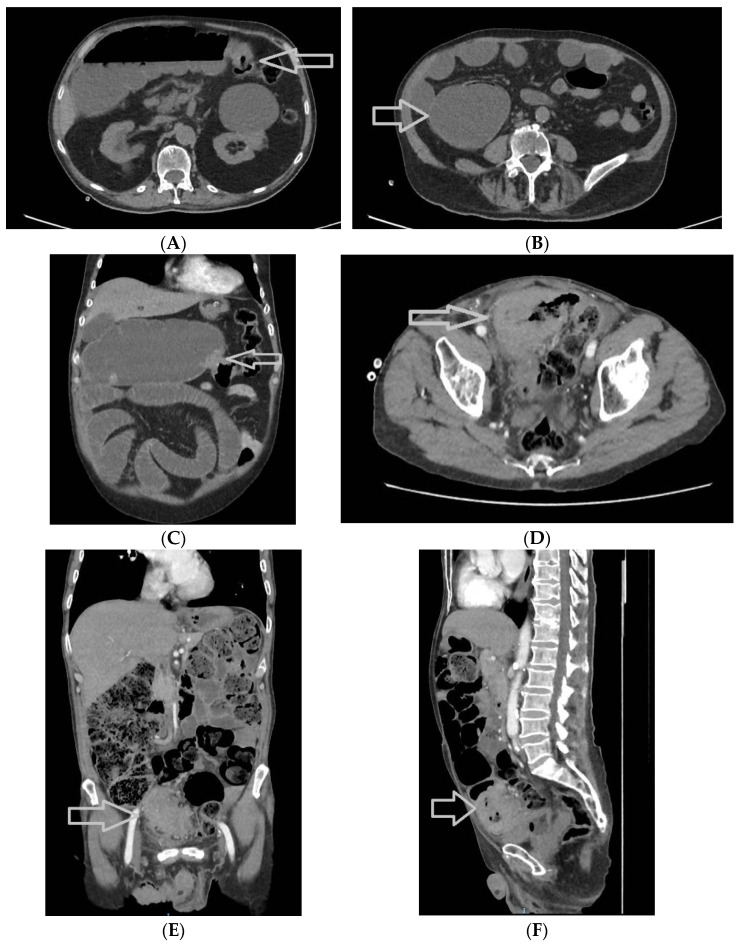

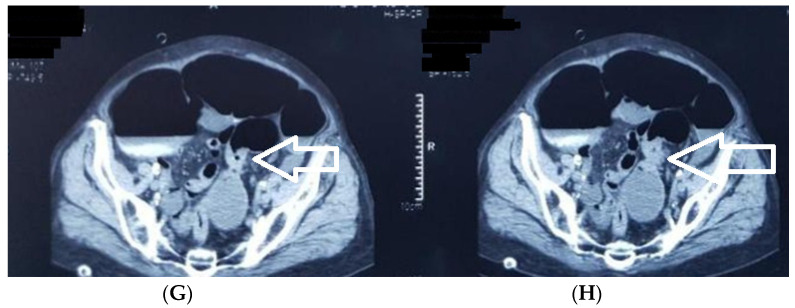
Abdominal and pelvic CT scans of colorectal tumors. (**A**) A 77-year-old male patient with a stenotic tumor on the transverse colon and a distended proximal colon, with air-fluid level, axial section (tumor highlighted with a white arrow); (**B**) The patient mentioned above, axial section—distended cecum with a high risk of perforation (white arrow); (**C**) The patient mentioned above, coronal section, tumor highlighted with a white arrow; (**D**) Another 77-year-old male patient with a sigmoid tumor with invasion in the urinary bladder, highlighted with a white arrow, axial section; (**E**) The same patient, coronal section, distension of the colon, sigmoid tumor highlighted with a white arrow; (**F**) The same patient, sagittal section, distension of the colon, sigmoid tumor highlighted with a white arrow; (**G**) A patient with a stenotic tumor of the colorectal junction (white arrow), with upper distension of the digestive tract; (**H**) The same patient, axial section; the CT scan reveals a distended colon and the tumor highlighted with a white arrow.

The patients who benefited from an emergency surgical procedure had an abdominal CT scan in 44 out of 69 cases (63.8%) (Figure 4A–H). In comparison, the patients who received an elective surgical procedure had an abdominal CT in 41 out of 84 cases (48.8%). We applied a Chi-square test, with a *p*-value of 0.073, just over the threshold of 0.05; therefore, there was no statistical correlation between an emergency surgical procedure and the preoperative abdominal CT scan.

**Figure 5:**
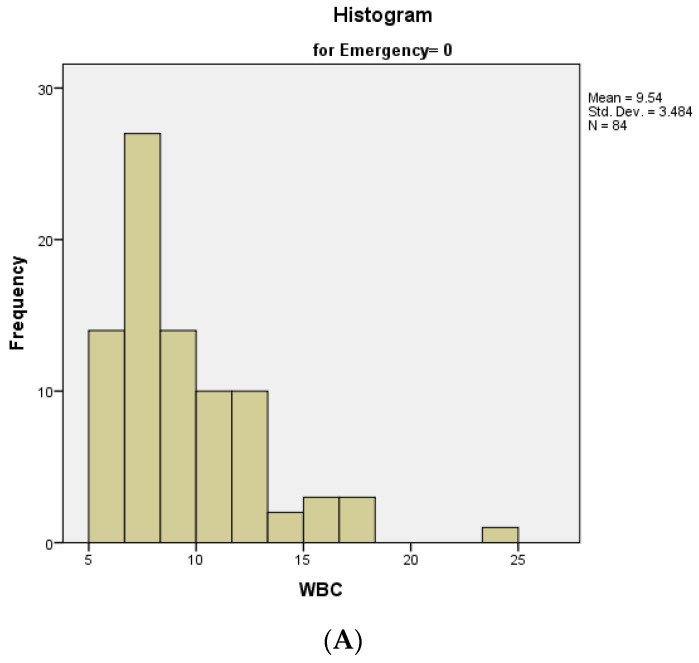

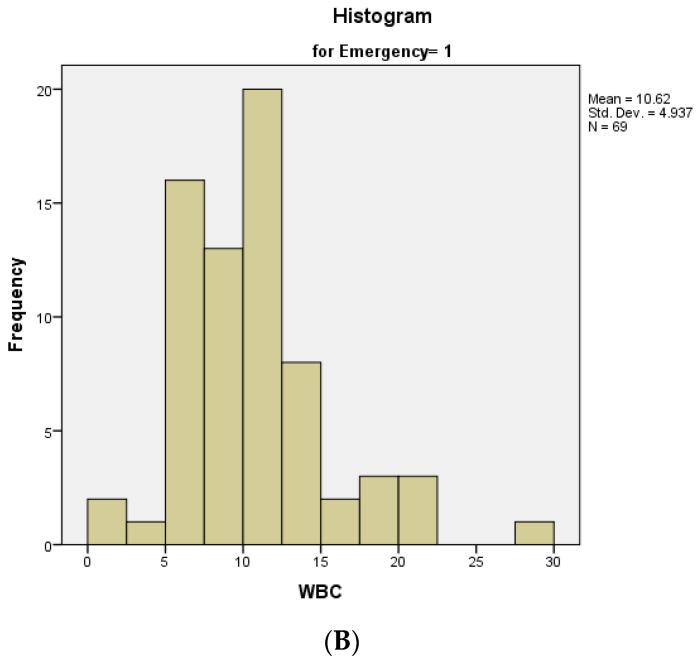
Distribution of WBC count: (**A**) In patients with an elective surgical procedure; (**B**) In patients with an emergency surgical procedure.

Preoperatively, a complete set of biological markers was determined for all the patients in the study. The WBC (white blood cell) count varied between 1000 and 30,000/mm^3^, with a mean value of 10,300/mm^3^ (normal values for our laboratory are 4800–10,800 WBC/mm^3^). We analyzed the distribution of the WBC count between the groups of patients who received or did not receive an emergency surgical procedure—both of the distributions were non-normal (Figure 5A,B). The median value of the WBC count for patients who received elective surgery was 8.67, with an interquartile range of 4, while the median value of the WBC count for patients who received emergency surgery was 10.00, with an interquartile range of 5. 

**Figure 6:**
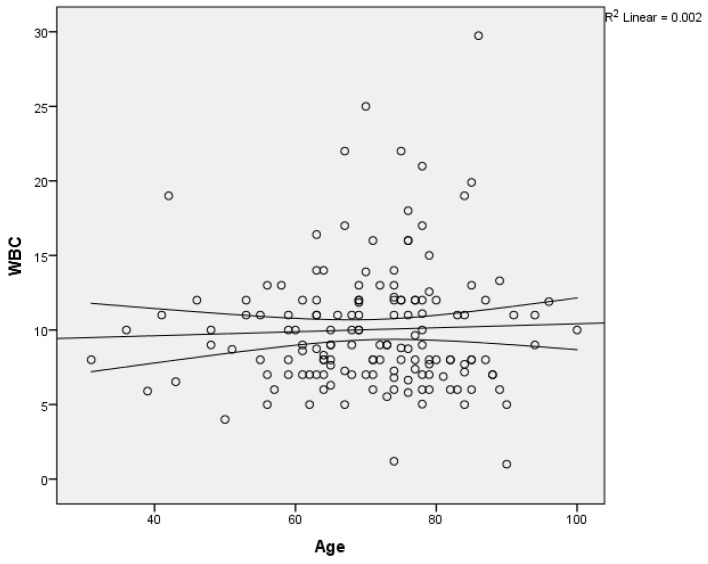
Scatter plot of WBC count on age with regression line and 95% mean confidence interval.

The relationship between the age of the patients in the study group and the WBC count was calculated using linear regression, without finding any linear relationship between the two variables (Figure 6). 

**Figure 7:**
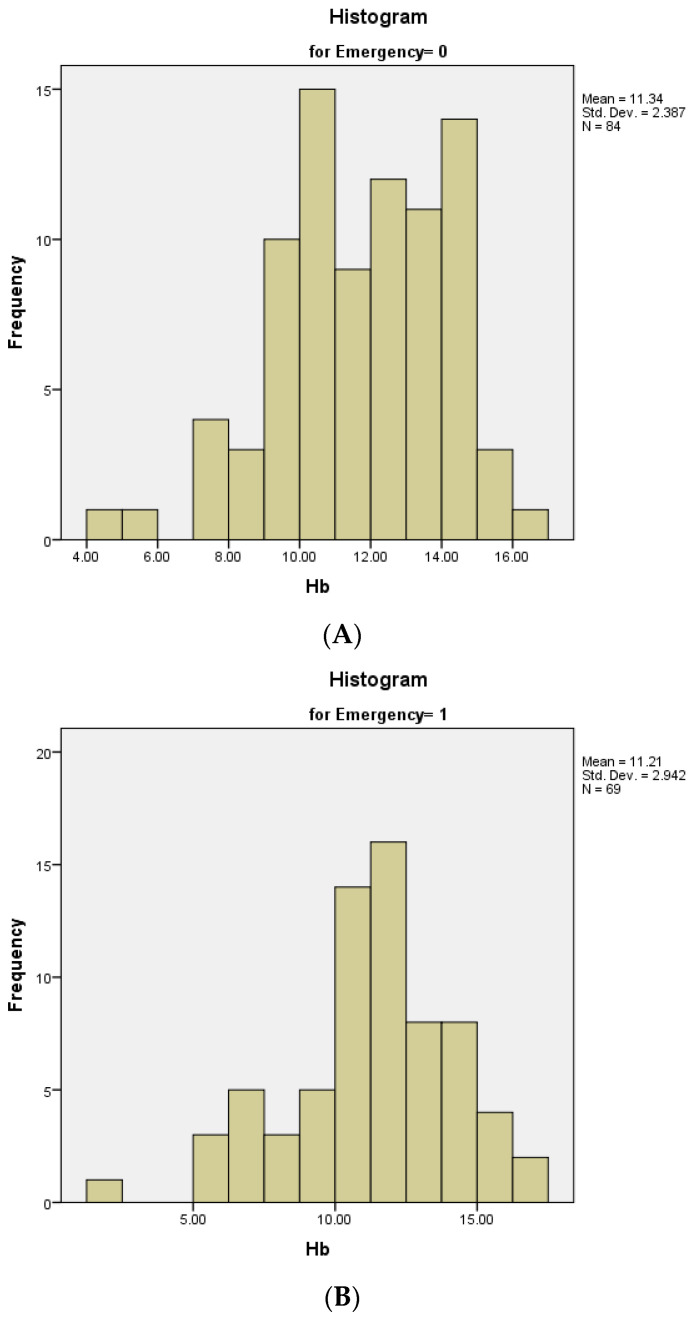
Distribution of Hb serum levels: (**A**) In patients with elective surgical procedure; (**B**) In patients with an emergency surgical procedure.

The Hb (Hemoglobin) levels varied between 2 g/dL and 17.00 g/dL, with a mean value of 11.28 (normal values for our laboratory are between 12–16 g/dL). For the patients receiving elective surgery the mean value was 11.33 g/dL, with a standard deviation of 2.38, while for patients who received emergency surgery, the mean value was 11.21 g/dL, with a standard deviation of 2.94. Both distributions were normal, with skewness and kurtosis values in the (−1;1) range (Figure 7A,B). 

Regarding gender, the mean value of Hb in women was 11.13 g/dL, with a standard deviation, of 2.53, while the mean value of Hb in men was 11.38, with a standard deviation of 2.73. Both distributions were also normal. Therefore, we performed a parametrical, two-sample t-test, with a *p*-value of 0.56, describing no statistical association of Hb values and gender in the patients in our group study.

Linear regression was used to analyze the relationship between the age of our patients and the preoperative Hb values, with a *p*-value of 0.248, above the 0.05 threshold. Also, linear regression was performed to determine if the preoperative Hb influenced the number of days of hospitalization. The *p*-value obtained was 0.181, so the null hypothesis was accepted, that is, there was no correlation between these two parameters. The mean Hb value for the patients who presented with lower GI bleeding was 10.77 g/dL.

In the case of preoperative glycemia, the maximum value in our study group was 117.57 mg/dL (normal values for our laboratory are 74–106 mg/dL). Both distribution among female and male patients were non-normal. For female patients, the median value was 106.50 and the interquartile range was 31, while for male patients the median value was 110 mg/dL, with an interquartile range of also 31. The non-parametrical test Mann Whitney U determined a *p*-value of 0.743. The distribution among patients who needed or not an emergency procedure was also non-normal, with a median for patients receiving emergency surgery of 116.93 mg/dL and a median for elective patients of 118.43, respectively. The interquartile range was 42 for patients receiving emergency surgery, while the interquartile range for elective patients was 26. The linear regression used to analyze the correlation between glycemia and age obtained a *p*-value of 0.096.

**Figure 8:**
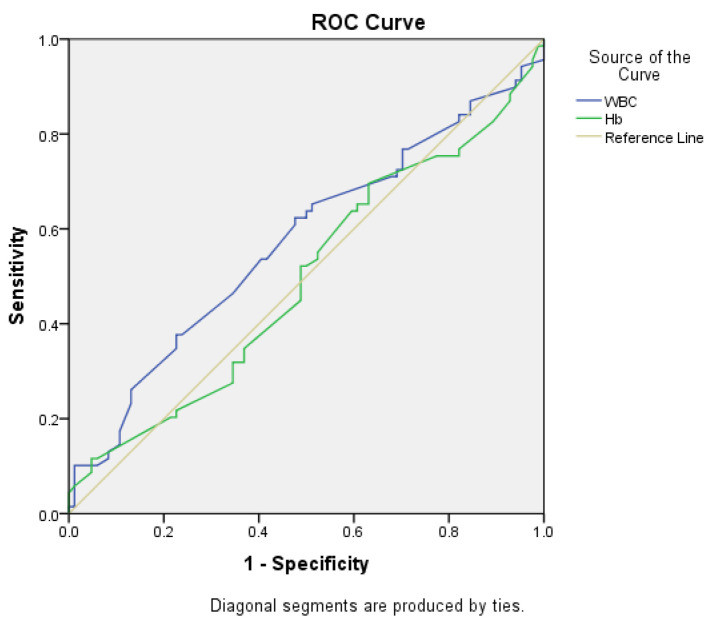
ROC curve used to determine the possibility of WBC count and Hb values as predictors for the necessity of an emergency surgical procedure.

We tried to determine if the preoperative values of WBC count and hemoglobin could predict the necessity for an emergency surgical procedure. A Receiver Operating Characteristic (ROC) curve was computed (Figure 8). Although the WBC count determined a higher value for the area under the curve in comparison to Hb (0.572 vs. 0.494), the *p*-values for the two variables were 0.124 and 0.895 respectively; therefore, neither of the two biological parameters was a significant predictor for the necessity of an emergency surgical procedure (the 95% confidence intervals contained the value 0.5). 

Further, the topography of colorectal cancer, measuring the frequency of these categories of patients regarding the type of surgical procedure (emergency/elective), was determined (Table 3). 

The most frequent topography of the tumor in our study group was the sigmoid colon, with 30 patients (19.60% of the study group), followed by the colorectal junction, with 17 patients (15.68% of the study group), and the superior rectum and inferior rectum, with 17 patients in each subcategory (11.11% of the study group). We performed a Chi-square test for each of the topographies concerning the type of surgery that was performed. The *p*-values obtained were all above the 0.05 threshold, except one. In the case of sigmoid colon topography, Fisher’s exact test indicated that there was a significant difference in the type of surgery between patients with or without sigmoid cancer. 

**Figure 9:**
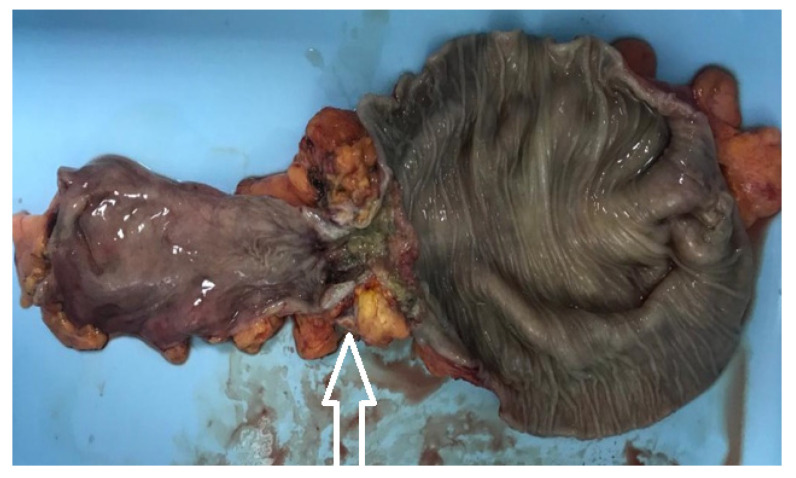
The postoperative aspect of the resected specimen—stenotic tumor of the left colon—pathology result of tubular adenocarcinoma (tumor highlighted with a white arrow).

**Figure 10:**
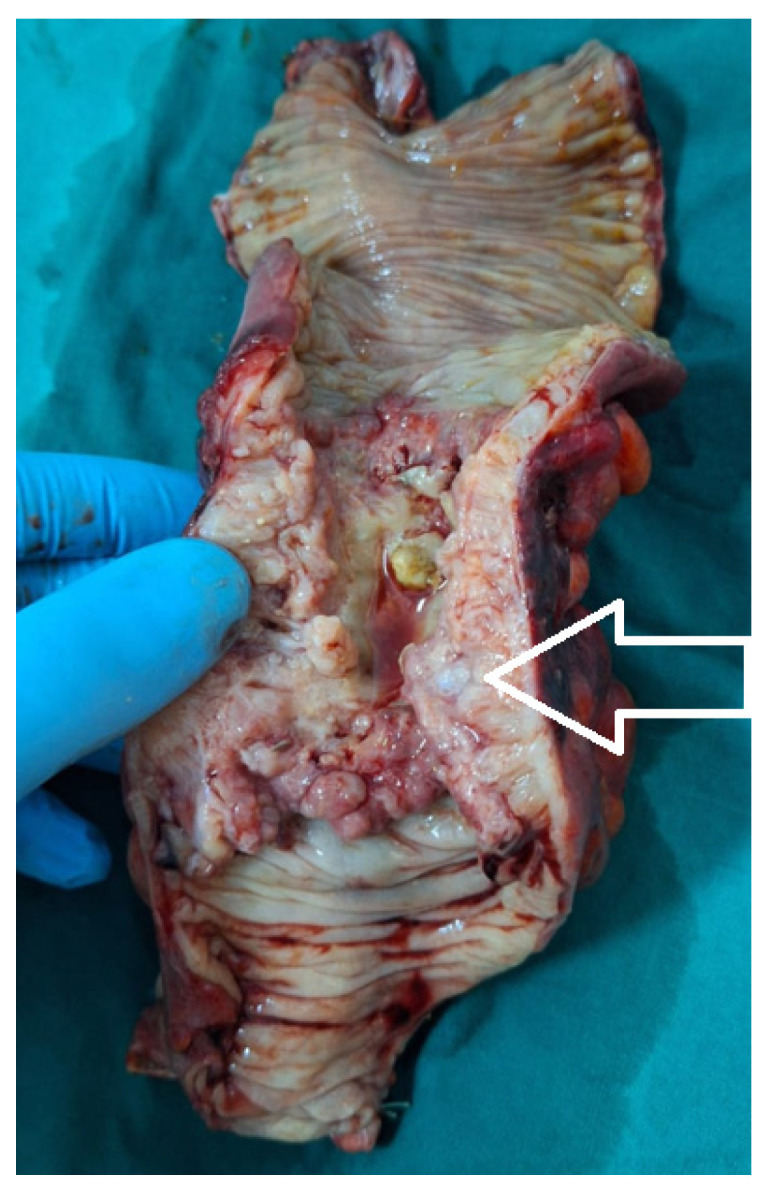
The postoperative aspect of a tubular adenocarcinoma of the colorectal junction (white arrow), with peritumoral abscess, perforation, and secondary generalized peritonitis.

The topographies of the tumors were grouped into three sub-categories:

- right colon (including the ileocecal valve, cecum, ascending colon, hepatic flexure, and transverse colon), consisting of 39 patients, 25.5% of the study group;
- left colon (including the splenic flexure, descending colon, and sigmoid colon), consisting of 47 patients, 30.7% of the study group (Figure 9);
- rectum (including the rectosigmoidian junction (Figure 10), superior rectum, medium rectum, inferior rectum and anus), consisting of 67 patients, 43.8% of the study group.

We designed a 2 × 3 Chi-square table (Table 4) that revealed a difference in percentages especially regarding the emergency procedure in the three topographical segments—66.0% for the left colon, compared to only 38.5% for the right colon and 45.1% for the rectum. Since every value from our cells was greater than 5, we retained the Pearson Chi-Square *p*-value of 0.002, which is statistically significant for a difference in the three topographical sub-categories regarding the type of surgery performed. 

A 2 × 3 Chi-square table relating the three topographical subcategories and the gender of the patients was also designed, but the *p*-value obtained from the Pearson Chi-Square test did not determine a statistical association between these two variables, with a *p*-value of 0.927.

**Figure 11:**
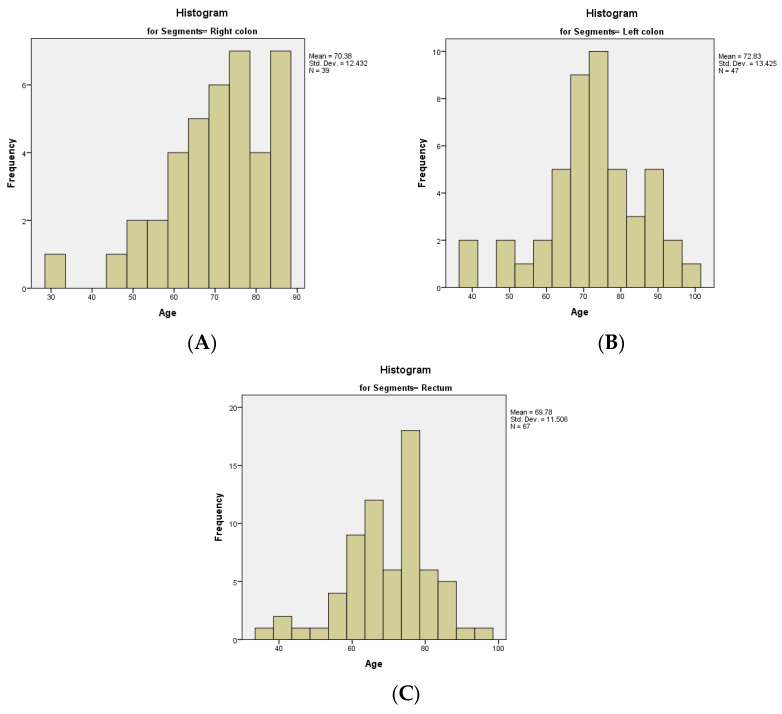
Distribution of age with various topographies of the tumor: (**A**) In the right colon; (**B**) In the left colon; (**C**) In the rectum.

The relationship between the age of the patients and the topography of the tumor was calculated. The age distribution for the right colon was non-normal and left-tailed, with a median of 71.00 years and an interquartile range of 17. The age distributions for the left colon and the rectum were normal, with a mean value of 72.83 years (95% CI) and a standard deviation of 12.432 for the left colon, respectively a mean value of 69.78 years (95% CI) and a standard deviation of 11.506 for the rectum (Figure 11A–C). We calculated the Spearman’s correlation coefficient, with a rho value of 0.498 and a strong relationship between age of the patients in the study group and the topography of the tumor.

We tried to determine the association between the topography of the tumor and the values of WBC count and Hb. The distribution of the biological markers was non-normal. The median value of WBC count was 8.7 × 10^3^/mm^3^ for the right colon, 10.0 × 10^3^/mm^3^ for the left colon, and 9.0 × 10^3^/mm^3^ for the rectum, respectively. The median value for Hb was 11.5 g/dL for the right colon, 12.0 g/dL for the left colon, and 11.0 g/dL for the rectum, respectively. We applied a non-parametrical test, Kruskall–Wallis, with both *p*-values for WBC and Hb being above the threshold, 0.452 and 0.359; therefore, the distribution of WBC and Hb levels was the same across the categories of patients with tumors in the three colorectal segments.

The number of hospitalization days was similar for the patients classified by the localization of the tumor—the median values of 12 days for the right colon, 11 days for the left colon, and 11 days for the rectum, respectively, with a non-normal distribution. The Kruskall–Wallis non-parametrical test determined a *p*-value of 0.716; therefore, the distribution of hospitalization days was the same across categories of patients with tumors in the three colorectal segments.

The type of surgical procedure that we performed can be seen in Table 5. The most frequent type of procedure was right hemicolectomy, in 33 patients (21.6% of the study group), followed by rectosigmoid resection, in 32 patients (20.9% of the study group) and tumoral biopsy, in 24 patients (15.7% of the study group). The biopsies were performed for rectal cancers and in 12 cases were associated with the creation of an upward ostomy on the sigmoid colon, as a first step in the oncological multimodal management of the rectal cancer, before radiotherapy. In our study group, the surgical procedure was finished by performing an anastomosis in 75 patients (49%), an ostomy in 66 patients (43.1%), and neither of the above-mentioned in 12 patients (7.8%), being about a tumoral biopsy. 

The relationship between the type of surgical procedure that was performed and the emergency regimen of the procedure was analyzed using a Chi-square test. The results can be seen in Table 5 and show a statistical association for the segmentary resections of transverse and sigmoid tumors, which were performed more frequently in emergency surgery, with *p*-values of 0.020 and 0.037, respectively. Also, we determined the relationship between the type of surgical procedure and the result of the procedure (ostomy, anastomosis, or neither) using a 2 × 3 Chi-square test. The results, which can also be seen in Table 5, determined *p*-values below the threshold for right hemicolectomy, which was finalized mostly with an anastomosis (*p* = 0.002), for segmentary resection of the sigmoid colon, which was finalized more frequently with an ostomy (*p* = 0.044), and for rectosigmoid resection, which was finalized more frequently with an anastomosis (*p* = 0.046). 

The influence of gender on the type of outcome of the surgical procedure (ostomy/anastomosis/neither) was determined. The 2 × 3 Chi-square test revealed similar frequency values, with no statistical association—a *p*-value of 0.882. 

Also, we tried to find out if the age of the patients is a risk factor for the outcome of the surgical procedure. The 12 patients who had neither an ostomy nor an anastomosis were eliminated. An ROC curve for the remaining subgroup of 141 patients was compiled, also without any statistical association, with a determined *p*-value of 0.238. For the same subgroup of 141 patients, we asked if the type of outcome could determine the number of hospitalization days. The distribution of the hospitalization days in relation to the binary variable of surgical outcome was non-normal, with a median of 11 days and interquartile range of 7 for patients with an ostomy, while for patients with an anastomosis had a median of 13 and an interquartile range of 6. Therefore, we performed a non-parametrical Mann–Whitney U Test that determined the *p*-value of 0.024, which was statistically significant, rejecting the null hypothesis and stating that the distribution of hospitalization days was not the same across patients that received an ostomy or anastomosis.

Early reintervention was performed in six patients. Early postoperative complications included wound abscess (17 patients), fistula of the anastomosis (three patients), abdominal evisceration (two patients), and stenosis of the ostomy in one patient. 

A Chi-square test was performed to determine if there was any correlation between the type of surgery performed (elective/emergency) and the development of a wound abscess, but a *p*-value of 0.800 was obtained.

In our study group, eight patients died, with a mortality of 5.22%. The mean age of the deceased patients was 69.75 years, and the median value was 70.50 years, with a minimum of 55 years and a maximum of 84 years. We created an ROC curve to determine the influence of the age of the patients on mortality, obtaining a *p*-value of 0.673. In addition, the correlation between the gender of the patients and mortality determined a *p*-value of 0.467.

The mean number of hospitalization days for the deceased patients was 13.50 days, with a median of 11.00, a minimum value of three days, and a maximum value of 39 days. 

Regarding the character of the surgical procedure, seven patients out of the eight patients that died received emergency surgery; the Chi-square test obtained a *p*-value of 0.023, which was statistically significant. There was no statistical association between the localization of the tumor (right colon, left colon, rectum) and mortality, with a *p*-value of 0.427.

The list of deceased patients, regarding age, gender, number of hospitalization days, type of surgical procedure performed, topography of the tumor, and surgical procedure can be seen in Table 6:

## 4. Discussion

Colorectal cancer remains one of the most frequent cancers in the world, with a heavy burden that involves high mortality, alterations in the quality of life of the patients and their families, and also the financial costs of the medical systems. 

Significant developments have been made in recent years regarding the understanding of the role of several risk factors involved in this disease, the genetic and molecular mechanisms of tumoral growth, and new therapeutic measures. 

In addition, great efforts are being made regarding screening methods and more personalized treatments that involve multidisciplinary teams to reduce the impact of colorectal cancer. 

According to research by the American Cancer Society, men have a 30% higher risk of developing colorectal cancer than women. Also, men who are diagnosed with colorectal cancer have 40% higher mortality and a worse prognosis than women [4,11]. However, women are more often diagnosed with cancer of the right colon, which tends to be found in more advanced stages of evolution and seems to be more aggressive than cancer of the left colon [12,13]. Mechanisms for these disparities are not fully understood, but theories might suggest that certain risk factors (alcohol consumption, tobacco), dietary patterns, and sex hormones might be involved [14]. 

In our study group, consisting of 153 patients, we accounted also for more male patients diagnosed with CRC than females, as many as 87 patients (56.9%). Furthermore, out of the eight patients from the study group that died, six were males (75%). 

The risk for colorectal cancer increases with age, being more frequent in patients over 50 years old. According to global statistics for the year 2018, the median age is 68 years for men and 72 years for women. The number of colorectal cancer cases increases gradually, being between 1.5–5.4 per 100,000 cases for the interval 25–39 years (1.4–5.3 in men and 1.6–5.5 in women), between 10.3–18.5 per 100,000 cases for the interval 40–49 years (10.2–18.9 in men 10.4–18.1 in women), between 34.3–62.5 per 100,000 cases for the interval 50–64 years (37.4–72.7 in men and 31.2–53.2 in women), between 92.6–212.2 per 100,000 cases for the interval 65–85 years (107.7–229.7 in men and 79.2–200.4 in women), and up to 240.9 in patients over 85 years (264.0 in men and 229.2 in women) [1]. 

Older age is considered one of the most important risk factors in the development of colorectal cancer, with approximately 90% of cases occurring in patients over 50 years old [4,15,16]. Other studies estimate that patients over the age of 65 years have a risk around three times higher to develop a CRC than patients with ages between 50–64 years and 30 times higher than patients with ages between 25–49 years [17]. The relationship between age and colorectal cancer is obvious in developed countries, where higher life expectancy determines an increased number of old people [18]. However, recent studies show that in Europe and the United States, the incidence of colorectal cancer has increased in younger groups of adults, between 20–49 years old; therefore, the recommendations for the screening of colorectal cancer start at the age of 50 years [19,20]. 

These data are similar to the results from our study group of 153 patients, in which the mean value of patients’ age was 70.87 years and the median was 72 years. The mean age regarding gender was similar, with a value of 70.85 years for women and 70.89 years for men, with no statistical significance between the two genders regarding age.

Costs involving colorectal cancer are extensive and account not only for the number of days of hospitalization with all the medical care involved but also the complementary oncological treatments and the impact of this disease on the quality of life of the patients and their families and the number of years lost due to the high number of related deaths. In our group, the mean value of hospitalization days was 12.61 days, with a median value of 11 days. The distribution of hospitalization days was non-Gaussian, with most of the values around the mean value, but there were a few higher values (with a maximum of 39 hospitalization days) due to important comorbidities, complications that occurred postoperatively, and the need for treatment in the intensive care unit. 

Clinical presentation of colorectal cancer may involve one of three pathways: asymptomatic patients (early CRC being usually asymptomatic [21]), suspicious symptoms and signs, or emergency presentation involving bowel obstruction, digestive perforation, or lower gastrointestinal bleeding. Most colorectal cancers—70% up to 90%—are diagnosed after the onset of the clinical manifestations. Symptoms of colorectal cancer usually appear due to the growth of the tumor in the lumen or due to the spread of the tumor to the surrounding structures. Therefore, most of the symptomatic cases are related to an advanced CRC [22,23]. Several large studies have revealed the frequency of the clinical manifestations in colorectal cancer. In a retrospective cohort of over 29,000 patients referred by their general practitioners to an outpatient colorectal surgery department over 22 years, the 1626 patients who were eventually diagnosed with CRC presented: change in bowel habits (74%), lower digestive bleeding (71%), rectal mass (24.5%), abdominal mass (12.5%), iron deficiency anemia (9.6%), and abdominal pain as a single symptom (3.8%) [24]. Another recent study that evaluated 388 patients diagnosed with CRC that determined a diagnostic colonoscopy presented lower digestive bleeding in 37% of the cases, abdominal pain in 34% of the cases, and anemia in 23% of the cases. Symptoms also vary on the location of the tumor. Bowel obstruction symptoms and changes in bowel habits suggest the topography of the left colon; anemia can raise the suspicion for a tumor of the right colon (cecal and ascending colon tumors can lead to four times higher blood loss than other colorectal tumors); and rectal tumors usually present tenesmus, rectal pain, and diminished caliber of stools. The pain can occur no matter the topography of the tumor and can be caused by intestinal perforation, obstruction, or peritoneal metastases [25,26,27]. 

In our group, the most common clinical manifestations were pain (73.2% of the study group), followed by abdominal distension (69.3% of the study group) and absence of intestinal transit (38.6% of the study group). The explanation for these values and the fact that pain was the main clinical manifestation was the large number of patients that presented to our department and required an emergency surgical procedure. The mean Hb value for the patients who presented with lower GI bleeding was 10.77 g/dL, slightly lower than the entire group of patients’ average. However, the low number of patients who presented with lower GI bleeding suggested that trying to make a correlation between these patients and the Hb value is highly predisposed to statistical error. Also, there was no statistical association between Hb values and type of emergency/elective surgery, gender, or age of the patients.

Despite efforts to implement screening methods, according to several studies, approximately 30–33% of patients with colorectal cancer will present with clinical manifestations requiring an emergency surgical procedure [28,29,30,31]. Emergency colorectal procedures are associated with higher morbidity and mortality rates than elective procedures [32,33,34]. Several studies have suggested that emergency colorectal resections are associated with poor oncological outcomes [33,34], due to a more advanced tumoral stage, higher histological grade, and a higher probability to present lympho-vascular invasion or metastases, but also due to physiological abnormalities or neglected comorbidities, characteristic of patients presenting an emergency [35,36,37]. On the other hand, some studies claim that emergency curative resection for colorectal cancer has similar outcomes regarding survival to elective surgical procedures [38]. 

In our study group, 69 patients received emergency surgery (45.1%), while 84 patients (54.9%) benefited from elective surgery. In all the cases we used thromboprophylaxis and antibiotic prophylaxis, according to the current guidelines [39]. We analyzed the correlation between the patients with colorectal cancer who benefited from an emergency procedure and several general parameters (age, gender, number of hospitalization days, or area of origin—urban/rural), but found no statistical association. We also tried to determine if the preoperative values of WBC count and hemoglobin levels could predict an emergency colorectal operation. However, both the *p*-values for the two variables were over the threshold; therefore, neither of the two biological parameters was a significant predictor for the necessity of an emergency surgical procedure. 

Although initial classifications of the topography of colorectal cancer define three regions—proximal colon, distal colon and rectum [40,41,42]—current definitions use the right colon (derived from the embryologic midgut) which includes the cecum, ascending colon, hepatic flexure, and the proximal two-thirds of the transverse colon, respectively; and the left colon (derived from the embryologic hindgut), which includes the distal third of the transverse colon, splenic flexure, descending colon, sigmoid colon, and rectum. Some classifications define the rectum as a separate entity [43]. Colorectal cancer is localized in the distal or left colon in approximately 70% of cases. However, several studies suggest that left-sided colorectal cancer has been observed less frequently and that cancer of the right colon has had an upward trend in recent years [44,45]. 

There are several well-documented differences between right and left colon cancer. The microbiome is different [46,47,48], and recently published data have shown that colorectal cancer-associated bacterial clusters are differentially correlated with mucosal gene expression profiles, while a few clusters are partially associated with the expression of pro-inflammatory genes in the mucosa, which may result in colorectal cancer in the future [49]. Several molecular and chromosomal differences have been reported [50,51,52,53,54], while chromosomal instability has been detected in approximately 30% of cases of right colon cancer and 70% of cases of left colon cancer [55,56]. The median age of patients with right colon cancer is higher compared to patients with left colon cancer, while a greater proportion of patients with right colon cancer are females [51,57,58]. Right colon cancer is likely to have a more advanced stage at the initial presentation compared with left colon cancer [51,57,59]. Also, right colon cancer often gives metastases to the peritoneum, while a greater proportion of left colon cancer cases will give metastases to the liver and lung [54]. 

In our study group, the most frequent topography of the tumor was the sigmoid colon, in 19.60% of the patients, followed by the colorectal junction, in 15.68% of the patients, and superior rectum and inferior rectum, in 11.11% of the patients in each subcategory. The same as the data from the literature, the frequency of cancer on the left side of the colorectal segment was higher. Statistical analysis indicated that there was a significant difference in the type of surgery (elective/emergency) between patients with or without sigmoid cancer, this type of disease being associated with an emergency surgical procedure. We grouped the patients into three categories regarding the topography of the tumor (right colon, left colon, rectum) and analyzed the types of surgical procedures for each category. Emergency surgical procedures were performed in 38.5% of the patients with right colon cancer, 66% of the patients with left colon cancer, and 34.3% of the patients with rectal cancer, obtaining a statistically significant *p*-value of 0.002. The relationship between age and topography showed a normal Gaussian distribution for patients with left colon cancer and rectal cancer and a left-tailed non-normal distribution for patients with right colon cancer. The statistical analysis showed a strong relationship between the age of the patients in the study group and the topography of the tumor. We also analyzed the relationship between the topography of the tumor and gender, number of hospitalization days, WBC count and hemoglobin levels at admission, but obtained no correlation among these parameters. 

The main “pillar” of the management of colorectal cancer is surgical resection, traditionally providing the only curative treatment [60,61]. The type of resection depends on the stage of the tumor. For T_0_ and T_1_ colorectal cancers, in the absence of prognostic factors that may indicate a high risk of lymph node involvement, endoscopic mucosal resection/submucosal dissection can be used for local excision of the tumor [62,63]. Where resection is performed, the location of the tumor and the corresponding vessels and lymph nodes define the segment that should be resected and the surgical procedure (e.g., right hemicolectomy, left hemicolectomy, segmentary colectomy of the transverse colon or sigmoid colon, proctocolectomy, abdominoperineal rectum amputation, etc.) [61]. The surgical principles of mesocolic and mesorectal dissection, described by Hohenberger et al. and Heald et al., respectively, have resulted in significant improvements in oncological outcomes [64,65]. 

Regarding non-surgical treatment, neoadjuvant radio-chemotherapy is used for locally advanced rectal cancer to increase the rate of complete resection and decrease the possibility of recurrence [66,67]. Furthermore, recent trials have proved that neoadjuvant chemotherapy can be used for a subgroup of patients with locally advanced, resectable colorectal cancer [68]. Adjuvant chemotherapy is recommended for patients with stage III colorectal cancer to reduce the risk of tumor recurrence. Also, systemic adjuvant therapy is used for patients with emergency presentation, anastomotic leakage, T_4_ tumors, inadequate node sampling, or poorly differentiated histology [69,70]. Recently, neoadjuvant immunotherapy has presented great results for patients with locally advanced or metastatic colorectal cancer, but only for a subgroup with a deficient MMR (Mismatch Repair) system. However, only about 15% of patients with colorectal cancer have this characteristic, most of them with cancers of the right colon, while for patients with rectum topography, this value drops under 5% [71,72,73]. 

Regarding our group of patients, the most frequent type of procedure was right hemicolectomy (21.6% of the study group), followed by rectosigmoid resection (20.9% of the study group), and tumoral biopsy (15.7% of the study group). The surgical procedure was finished by performing an anastomosis in 49% of the patients and an ostomy in 43.1% of the patients, while for 7.8% of the patients, a tumoral biopsy was performed. The segmentary resections of the transverse and sigmoid colon were statistically associated with an emergency surgical procedure, with *p*-values of 0.020 and 0.037, respectively. Also, statistically significant associations were determined for right hemicolectomy, which was finalized mostly with an anastomosis (*p* = 0.002); for segmentary resection of the sigmoid colon, which was finalized more frequently with an ostomy (*p* = 0.044); and for rectosigmoid resection, which was finalized more frequently with an anastomosis (*p* = 0.046). There was no statistical association between surgical outcome (anastomosis/ostomy/biopsy) and gender or between the age of the patients as a risk factor and the surgical outcome. However, we obtained a correlation between the number of hospitalization days and the type of surgical outcome (a median value of 13 days for patients with anastomosis and a median value of 11 days for patients with an ostomy), with a *p*-value of 0.024. 

Colorectal cancer is the third most common cancer and the second cause of death related to cancer worldwide [2]. In 2020, there were an estimated 930,000 deaths due to colorectal cancer and approximately 1.9 million new cases in the world. Projections suggest that by the year 2040 there will be 3.2 million new cases of CRC and 1.6 million deaths related to CRC each year, most cases predicted to occur in high or very high human development index (HDI) countries [3]. Several studies suggest that this increase is a result of environmental changes, like a more sedentary lifestyle, obesity, consumption of highly processed food, red meat consumption, alcohol, and an increase in overall life expectancy [4,74,75]. 

In our group, we had a mortality of 5.22%. The mean age of the deceased patients was 69.75 years, and the median value was 70.50 years, with a minimum of 55 years and a maximum of 84 years. There was no statistical association between age or gender and mortality, with *p*-values of 0.673 and 0.467, respectively. Furthermore, we determined that seven out of the eight patients who died in our group benefited from emergency surgery, which was statistically significant, with a *p*-value of 0.023. On the other hand, tumor topography did not influence mortality, with a *p*-value of 0.427. 

## 5. Conclusions

Our study found no statistical association between the regimen of the surgical procedure (emergency or elective) and age, hospitalization days, gender, or living area (urban/rural).

Regarding biological parameters, we found that there was no statistical association between the age of the patients in our group and white blood cell count or preoperative glycemia. Also, there was no association between preoperative hemoglobin values and gender or the number of hospitalization days. In addition, we determined that neither white blood cell count or hemoglobin values on admission were a significant predictor for the necessity of an emergency surgical procedure.

We determined that the topography of colorectal cancer is associated with the necessity of an emergency surgical procedure, especially for sigmoid tumors. The topography was also correlated with the age of the patients, but not with their gender, preoperative white blood cell count, or number of days of hospitalization. 

In the case of surgical procedures, our study also found that right hemicolectomy and rectosigmoid resection were finalized with an anastomosis rather than an ostomy, while the segmentary resection of the sigmoid colon was associated with an ostomy. There was no influence of the gender or age of the patients on the outcome of the surgical procedure (ostomy/anastomosis), while patients who benefited from an anastomosis had a longer hospitalization compared with the subgroup that received an ostomy. 

Finally, mortality was not associated with age or the topography of the tumors. Instead, it was correlated with the type of presentation, being found mostly in patients receiving emergency surgery. 

## Figures and Tables

**Table 1:** Correlation between type of presentation and several general parameters of the study group.

Type of Variables Analyzed	Type of Statistical Test	*p*-Value
Emergency vs. Age	Two-sample *t*-test	0.733
Emergency vs. Hospitalization days	Mann–Whitney U test	0.823
Emergency vs. Gender	Chi-square test	0.414
Emergency vs. Urban/Rural	Chi-square test	0.497

**Table 2:** Distribution of clinical manifestations of the study group on admittance (%).

		Preoperative CT Scan		
		No	Yes	Total
**Emergency** **procedure**	**No**	43	41	84
	**Yes**	25	44	69
	**Total**	68	85	153

**Table 3:** Distribution of patients with colorectal cancer regarding their topography, their type of surgery, and the statistical association.

Topography	Number of Patients	Number of Patients by Type of Surgery (Em = Emergency; El = Elective)	*p*-Value
Ileocecal valve	13	Em = 7; El = 6	0.568
Cecum	3	El = 1; Em = 2	1.000
Ascending colon	8	Em = 1; El = 7	0.074
Hepatic flexure	6	Em = 1; El = 5	0.223
Transverse colon	9	Em = 5; El = 4	0.732
Splenic flexure	8	Em = 6; El = 2	0.141
Descending colon	9	Em = 4; El = 5	1.000
Sigmoid colon	30	Em = 21; El = 9	**0.004**
Rectosigmoidian junction	24	Em = 10; El = 14	0.824
Superior rectum	17	Em = 7; El = 10	0.800
Medium rectum	9	Em = 2; El = 7	0.186
Inferior rectum and anus	17	Em = 3; El = 14	0.072

**Table 4:** 2 × 3 Chi-square table analyzing the relationship between the localization of the tumor and the type of surgery performed.

		Type Surgery			*p*-Value
		Elective	Emergency	Total	
**Topographical segment**	**Right colon** **% within right colon**	24 61.5%	15 38.5%	39 100.0%	**0.002**
	**Left colon** **% within left colon**	16 34.0%	31 66.0%	47 100%	
	**Rectum** **% within rectum**	44 65.7%	23 34.3%	67 100%	
	**Total** **% within total**	84 54.9%	69 45.1%	153 100%	

**Table 5:** Distribution of surgical procedures in the study group and the statistical association with the type of emergency regimen and the result of surgical procedure.

		Type of Surgery				Result of Surgical Procedure				
		Elective	Emergency	Total	*p*-Value	Ostomy	Anastomosis	Neither	Total	*p*-Value
**Type of surgical procedure**	**Right hemicolectomy**	22	11	33	0.167	8	25	0	33	**0.002**
	**% within**	66.7%	33.3%	100%		24.2%	75.8%		100%	
	**Left hemicolectomy**	11	13	24	0.367	10	14	0	24	0.253
	**% within**	45.8%	54.2%	100%		41.7%	58.3%		100%	
	**Segmentary resection of transverse colon**	3	10	13	0.020	5	8	0	13	0.440
	**% within**	23.1%	76.9%	100%		38.5%	61.5%		100%	
	**Segmentary resection of sigmoid colon**	7	14	21	0.037	14	7	0	21	**0.044**
	**% within**	33.3%	66.7%	100%		66.7%	33.3%		100%	
	**Rectosigmoid resection**	21	11	32	0.231	11	21	0	32	**0.046**
	**% within**	65.6%	34.4%	100		34.4%	65.6%		100%	
	**Upward ostomy on sigmoid colon**	7	5	12	1.000	7	0	0	7	-
	**% within**	58.3%	41.7%	100%		100%			100%	
	**Abdominoperineal resection**	6	2	8	0.295	8	0	0	8	-
	**% within**	75%	25%	100%		100%			100%	
	**Tumoral biopsy**	17	7	24	0.118	12	0	12	24	-
	**% within**	70.8%	29.2%	100%		50%		50%	100%	

**Table 6:** General characteristics of deceased patients.

Number	Age	Gender M = Male, F = Female)	Hospitalization Days	Emergency (Em)/Elective (El)	Topography of the Tumor	Surgical Procedure
1.	74	M	13	Em	Sigmoid colon	Colectomy with ostomy
2.	67	M	4	Em	Descending colon	Left hemicolectomy with ostomy
3.	84	M	39	Em	Transverse colon	Colectomy with ostomy
4.	63	M	12	Em	Splenic flexure	Colectomy with ostomy
5.	78	F	21	Em	Colorectal junction	Left rectohemicolectomy with ostomy
6.	55	F	10	El	Colorectal junction	Rectosigmoid resection with ostomy
7.	78	M	3	Em	Transverse colon	Extended right hemicolectomy with ileostomy
8.	59	M	6	Em	Sigmoid colon	Colectomy with ostomy

## Data Availability

Data can be available upon request to the first author.
